# Alginate/Chitosan-Based Hydrogel Film Containing α-Mangostin for Recurrent Aphthous Stomatitis Therapy in Rats

**DOI:** 10.3390/pharmaceutics14081709

**Published:** 2022-08-16

**Authors:** Tiana Milanda, Faradila Ratu Cindana Mo’o, Ahmed Fouad Abdelwahab Mohammed, Khaled M. Elamin, Gofarana Wilar, Ine Suharyani, Nasrul Wathoni

**Affiliations:** 1 Department of Pharmaceutical Biology, Faculty of Pharmacy, Universitas Padjadjaran, Bandung 45363, Indonesia; 2 Department of Pharmaceutics and Pharmaceutical Technology, Faculty of Pharmacy, Universitas Padjadjaran, Bandung 45363, Indonesia; 3 Department of Pharmaceutics, Phaculty of Pharmacy, Minia University, Minia 61519, Egypt; 4 Global Center for Natural Resources Sciences, Faculty of Life Sciences, Kumamoto University, Kumamoto 862-0973, Japan; 5 Department of Pharmacology, Faculty of Pharmacy, Universitas Padjadjaran, Bandung 45363, Indonesia; 6 Department of Pharmaceutics, School of Pharmacy Muhammadiyah Cirebon, Cirebon 45153, Indonesia

**Keywords:** recurrent aphthous stomatitis, α-mangostin, chitosan, alginate, hydrogel film

## Abstract

Recurrent aphthous stomatitis (RAS) is a prevalent clinical disorder that causes mouth ulcers. Furthermore, corticosteroid treatment has been widely utilized for RAS therapy; however, it has side effects on the oral mucosa that limit its application. This study aimed to develop a novel RAS therapy with the natural ingredient α-mangostin, delivered by alginate and chitosan polymers-based hydrogel film (α-M Alg/Chi-HF). To prepare α-M Alg/Chi-HF, the solvent evaporation and casting methods were used, then characterized by using SEM, FTIR, and XRD. Based on the characterization studies, the α-M in α-M/EtOH Alg/Chi-HF with ethanol (EtOH) was found to be more homogenous compared to α-M in Alg/Chi-HF with distilled water (H_2_O) as a casting solvent. The in vitro viability study using NIH3T3 cells showed 100% viability of α-M Alg/Chi-HF (EtOH) and Alg/Chi-HF after 24 h incubation, indicating well tolerability of these hydrogel films. Interestingly, the in vivo studies using male white rats (*Rattus norvegicus* Berkenhout) proved that α-M/EtOH Alg/Chi-HF with a recovery of 81.47 ± 0.09% in seven days significantly more effective RAS therapy compared to control. These results suggest that α-M/EtOH Alg/Chi-HF has the potential as an alternative for RAS therapy.

## 1. Introduction

The mucous layer in the oral cavity has the main function of protecting the inside of oral cavity tissue from external injury by resisting abrasion caused by daily normal activities [1]. Apart from protecting against mechanical injury, mucous membranes can control the growth of infection-causing bacteria when they enter and spread into tissues. Its high activity puts the oral mucous membrane at risk of injury. A common injury is the formation of ulcers in the oral mucosa, which is known as recurrent aphthous stomatitis (RAS). Besides occurring in the buccal mucosal layer, RAS also often occurs on the tongue and gums in the oral cavity [2]. RAS is characterized by the appearance of reddish to white lesions with a round shape on the tongue, gums, and buccal areas. Based on the size of the lesion, RAS is divided into three categories: major RAS, minor RAS, and herpetiform [3]. RAS is known for the formation of lesions in the oral cavity caused by several factors, including hormonal changes, trauma, drug use, food hypersensitivity, stress, and hereditary factors. In addition, trauma will induce edema, which in turn increases the viscosity of the extracellular matrix of the oral mucosa [4,5].

RAS therapy focuses on inhibiting the inflammatory reaction and regenerating epithelial cells. The most commonly used drugs for RAS therapy are corticosteroids. However, their use results in several side effects, such as discoloration of the oral mucosa. In addition, corticosteroids can also trigger cutaneous bacterial complications and ongoing infection. The main mechanism of action of corticosteroids in RAS therapy only blocks inflammatory reactions but does not contribute to the regeneration of epithelial cells, so they are less efficient in the treatment of RAS [5]. Referring to these shortcomings, it is deemed necessary to develop RAS therapy in order to provide more effective and efficient results with minimal side effects. Natural ingredient-based therapy can be used as an alternative to corticosteroids. Mangosteen (*Garcinia mangostana* L.) has the active xanthone metabolite “α-mangostin/α-M”, which has been studied to have pharmacological activity in wound healing, inhibiting inflammatory reactions, and regenerating epithelial cells [6].

Mangosteen rind contains a variety of xanthones, including α-M, so that it can be applied to the therapy of RAS. To increase the efficiency and effectiveness of α-M, it was formulated into a hydrogel film dosage form with a transdermal delivery system. This aims to increase the percent absorption of the active substance and increase the contact time on the mucous membrane. In a previous study by Wathoni et al. (2019), a hydrogel film formulation of α-M was carried out for RAS therapy, where the α-M was dissolved in methanol; as it is well known, the use of methanol as an organic solvent can cause significant toxicity [7]. Therefore, in this research, the α-M was dissolved in ethanol rather than methanol, which is considered safer than methanol, and casting to a hydrogel film has been developed. In a previous study, the safety and effectiveness of the α-M hydrogel film have not been evaluated on experimental animals [7].

The hydrogel film is composed of several polymers used to entrap α-M and also to increase the consistency and elasticity of the hydrogel film. Chitosan and alginate were utilized as polymers, with plasticizers glycerin and propylene glycol. In our previous work, we found that alginate mixed with chitosan can be effectively fabricated into a hydrogel film using α-M, but it still needs to enhance its physicochemical qualities. The safety and efficacy of RAS treatment should also be evaluated [7]. Therefore, in this study, alginate/chitosan-based hydrogel film containing α-M (α-M Alg/Chi-HF) was modified using ethanol to dissolve α-Mangostin. Furthermore, the safety and effectiveness of α-M Alg/Chi-HF were evaluated in vitro using NIH3T3 cells and in vivo using male Wistar rats (*Rattus norvegicus*), respectively.

## 2. Materials and Methods

### 2.1. Materials

The α-mangostin (α-M) was obtained from Chengdu Biopurify Phytochemicals Ltd. (Chengdu, China). Chitosan (Chi) and sodium alginate (Alg) were obtained from Sigma Aldrich^®^ (PT. Elo Karsa Utama, Bandung, Indonesia) and Wako^®^ (Jakarta, Indonesia), respectively. Propylene glycol, glycerin, pure ethanol, as well as all reagents were analytical classes and were used without further purification.

### 2.2. Methods

#### 2.2.1. Preparation of Alg/Chi HF, α-M Alg/Chi HF, and α-M/EtOH Alg/Chi HF

Alginate/chitosan-based hydrogel film (Alg/Chi-HF) was prepared with the solvent evaporation method [7]. At the initial stage, 0.5 g of sodium alginate was swelled in 25 mL of distilled water. Moreover, 0.5 g chitosan powder was swelled in 25 mL diluted acetic acid (0.5 mL acetic acid glacial in 24.5 mL distilled water). Alg was added slowly into Chi and mixed using a magnetic stirrer at a constant speed at 45 °C to obtain a homogenous mixture. The mixture was then supplemented with propylene glycol and glycerin. After mixing, homogenization with Ultra turrax (homogenizer) was carried out at a speed of 20,000 rpm for 10 min. To remove air bubbles from the mixture, it was centrifuged at 4500 rpm for 15 min. This mixture was then poured and leveled in a propylene box with a size of 5 × 5 × 1 cm^3^ and thickness of 2–4 mm, then dried at 60 °C for 24 h in an oven to obtain a hydrogel film. The purpose of drying at a temperature of 60 °C is to evaporate the solvent so that the alginate and chitosan molecules can effectively form a film [7,8,9].

Alg/Chi HF contains only a combination of polymer and plasticizer. The α-M Alg/Chi HF formula is composed of a combination of polymer, plasticizer, and α-M, which was directly dispersed in the mixture. The α-M/EtOH Alg/Chi HF is composed of a combination of polymer, plasticizer, and ethanol solution of α-M cast on the surface of the film (Table 1). The dose of α-M used was 250 μg, which refers to several studies regarding α-M as an anti-inflammatory [9].

**Figure 1:**
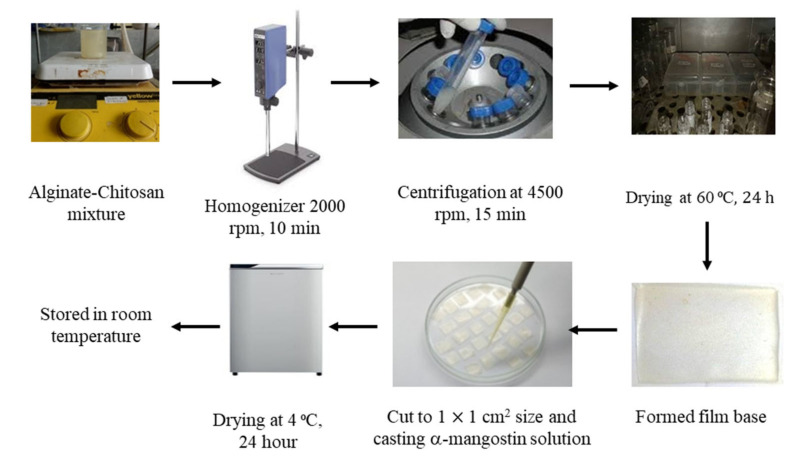
Fabrication and casting of hydrogel films.

After forming a thin film, 10 μL solution of α-M (2.5 mg/mL in ethanol) was cast on the surface of the 1 × 1 cm^2^ Alg/Chi-HF, then dried at 4 °C for 24 h (Figure 1). After drying, the hydrogel film was removed and stored in a dry place at room temperature [7,8,9].

#### 2.2.2. Determination of Thickness and Weight

The film thickness was measured by using the Teclock SM-112 Gauge; meanwhile, the weight was carried out by weighing the 1 × 1 cm^2^ hydrogel film using an analytical balance (Mettler Toledo PL303, Columbus, OH, USA). All measurements were carried out in triplicates, then the thickness and weight average and deviation of the hydrogel film of each formula were calculated [7,10].

#### 2.2.3. pH Measurement

pH examination was done using a pH meter (Mettler Toledo). Briefly, 1 g of the hydrogel films was dissolved in 10 mL distilled water, then the electrode was dipped in the solution, and the measurement was recorded. Measurement of hydrogel film pH was performed in triplicates to minimize bias; 1 g of the hydrogel films was dissolved in 10 mL distilled water then the electrode was dipped in the solution, and the measurement was recorded [11,12].

#### 2.2.4. Swelling Ratio

Swelling index testing was performed to find out the swelling characteristics of α-mangostin hydrogel film with alginate-chitosan polymers. The prepared hydrogel film sheet was weighed in advance (W_s_) and then soaked into phosphate-buffered saline (PBS) for 2 days at 37 °C. A completely expanded hydrogel film sheet was ejected. Excessive PBS on the surface was gently removed, then incubated at room temperature until it became constant (W_d_). The swelling ratio was calculated following the equation [13,14,15]:

(1) $$\mathrm{Swelling}\;\mathrm{Ratio}=\frac{\left(W_{d\;}-W_{s}\right)}{W_{s}}\times 100\%$$

#### 2.2.5. In Vitro Mucoadhesive Time

Evaluation of mucoadhesive time aimed to determine the time required by α-mangostin hydrogel film to adhere to the mucosal layer. This test was performed using the white rat’s mucosa. Each rat mucosal membrane was glued to the glass of the object using cyanoacrylate glue. One side of the film was moisturized with 1 mL of phosphate pH 6.8 and attached to the mucosa by pressing using a finger for 20 s. The mucosa and films that smeared on the glass object were hung and dipped into a cup containing 200 mL of 6.8 phosphate solution (temperature 37 °C ± 0.5 °C) and then stirred at a speed of 50 rpm to simulate the oral cavity environment. The time when the hydrogel film detaches was recorded as mucoadhesive time [7].

#### 2.2.6. X-ray Diffraction (XRD) Analysis

XRD analysis was carried out by placing a hydrogel film (2.5 × 2.5 mm^2^) on the sample holder of an X-ray diffractometer. X-ray diffraction patterns were obtained under conditions with Cu-Kα radiation filtered with Ni, voltage 40 kV, current 20 mA, divergent slit 10 mm (0.5°), scanning speed 5°/min, opened scattering, and receiving slit. Tests were carried out on a hydrogel base film as a control [7,16,17].

#### 2.2.7. Scanning Electron Microscopy (SEM) Analysis

The SEM analysis was carried out to determine the surface characteristics of the hydrogel films. A piece of Alg/Chi-HF, α-M Alg/Chi-HF, and α-M/EtOH Alg/Chi-HF were placed on aluminum stubs and coated with gold for 10 s (30 mM, 8 Pa). The hydrogel film was analyzed by SEM (JEOL JSM-6510LA Series, Tokyo, Japan) at a magnification of 5000× [7,8,9].

#### 2.2.8. Fourier Transform Infrared Spectroscopy (FTIR)

FTIR spectrophotometry was used to determine the structure of the functional group of a compound. The FTIR spectrum was obtained using an FTIR spectrophotometer (IR-Prestige-21). About 2 mg sample was blended with KBr and pressed to form a transparent pellet. Then the pellet was placed on the sample holder. FTIR characterization studies were performed in the range of wavenumbers 500–4500 cm^−1^ [13].

#### 2.2.9. In Vitro Drug Release

This test aimed to determine the release of α-M from hydrogel film polymers. The hydrogel film of 1 × 1 cm^2^ was soaked into 20 mL of PBS with a pH of 7.4 at 37 °C ± 5 °C and stirred at a speed of 50 rpm. Then, 5 mL of the solution was withdrawn from the media and replaced with 5 mL of fresh PBS every 15 min until the 260th minute. Samples were filtered with a 0.45 μm membrane, then diluted into several concentrations and analyzed using a UV-vis spectrophotometer at a maximum wavelength of 316 nm [7,18]. The kinetic data that had been obtained were then calculated using the Higuchi kinetic equation to determine the mechanism for the release of α-M with the following formula:

(2) Q=K×t12

Description:

Q = Drug Release against Time (%)
K = Value of Kinetic Constant

The Q value determines the drug release mechanism. When Q approaches 0.5, then the release mechanism follows Fick’s First Law of Diffusion, of which the diffusion value is 0.5 < Q < 1 (0.51–0.99) [8,19].

#### 2.2.10. In Vitro Cell Viability

A viability test was conducted to determine the safety of each hydrogel film formula on NIH3T3 cells. This test was carried out by seeding NIH3T3 cells (1 × 10^5^ cells/well) in 24 wells for 24 h; after reaching 80%, confluency cells were incubated by gently immersing each hydrogel film formula for 4 h in cell culture media. The control wells contain only cell culture media. Then, cells were rinsed twice with PBS, and 30 µL of WST-8 reagent was added and finally incubated at 37 °C for 30 min. The absorbance of the solution was measured at a wavelength of 450 nm, with a reference wavelength at 650 nm using the Tecan Infinite 2000 Spectrophotometer [7].

#### 2.2.11. In Vivo Ulceration Study

The in vivo evaluation was carried out by making ulcers on the tongue against the rats and then treated using hydrogel film. An ulcerative healing study was carried out to see the effectiveness examined in vivo in experimental animals. Histopathological changes that occurred were observed for a span of 7 days (the experimental animals were euthanized for evaluation on the 3rd, 5th, and 7th days).

An ulcerative healing study was carried out to see the effectiveness examined in vivo in experimental animals. The initial stage was selecting rats (*Rattus norvegicus*) with the characteristics of white rats, weighing 150–250 g, male, and maximum age of 6 months. The next stage was to quarantine or environmental adjustment for 7 days. The rats were divided into 4 groups, namely the negative control group (NC), positive controls group (PC), base group (Chi HF), and treatment group (α-M/EtOH Alg/Chi HF). Each group consists of 6 rats. The test was started by creating lesions on the rat tongues. Briefly, circular filter papers soaked with 15 µL of 50% acetic acid were attached to the rats’ tongues for 30 s to create round ulcers. Lesions would normally form after 24 h of the initial application. After the formation of lesions, the hydrogel film was applied to each group, in which the negative control group was not treated while the positive control group was given triamcinolone gel. The treatment was carried out for 7 days. Clinical healing of each ulcer was evaluated by measuring at each observation interval on the 3rd, 5th, and 7th day. Thus, wound healing was defined as a decrease in the ulcer area [20,21,22]. The tendency of linear regression for the ulcer area was analyzed using digital photography and the ImageJ^®^ application (https://imagej.nih.gov/ij/ (accessed on 11 April 2021)).

(3) $$\mathrm{Percent}\;\mathrm{recovery}\;\mathrm{of}\;\mathrm{ulcer}\;\mathrm{area}=\frac{\left(\mathrm{Ulcer}\;\mathrm{area}\;\mathrm{at}\;\mathrm{the}\;\mathrm{initial}-\mathrm{ulcer}\;\mathrm{area}\;\mathrm{at}\;\mathrm{the}\;t\;\mathrm{day}\right)}{\mathrm{Ulcer}\;\mathrm{area}\;\mathrm{at}\;\mathrm{the}\;\mathrm{initial}}\times 100\%$$

#### 2.2.12. Histopathological Analysis

Histopathological analysis was carried out by taking the affected rats’ tongues. The tongues were then soaked in 10% Neutral Buffered Formalin (NFB) to prevent spoilage. Then, the tissues were then blocked with paraffin and cut at a thickness of 3–5 µm using a microtome (Leica RM 2235, United States). The slices were processed for immunohistochemistry using Hemotoxyllin-eosin (HE) stains and then analyzed using an Olympus microscope. The parameters investigated were the formation of the epithelial layer in the tissue [20,21,23]. Epithelial thickness was measured by using the ImageJ^®^ application (https://imagej.nih.gov/ij/ (accessed on 11 April 2021)) [22].

#### 2.2.13. Data Analysis

The data from the qualitative characterization results were presented in the form of mean ± standard error of the mean (S.E.M.). The statistical comparison was performed using the Scheffe test. The value *p* < 0.05 was considered statistically significant. One-way ANOVA evaluation with Scheffe test using Statview was performed to evaluate the differences between the test group and the control group in in vivo. The statistical plots with Statview were described for studying the a-mangostin activities. The results are considered significant when *p* < 0.05 [21].

## 3. Results

### 3.1. Preparation of Alg/Chi HF, α-M Alg/Chi HF, and α-M/EtOH Alg/Chi HF

The concentrations of alginate and chitosan refer to previous studies where the most optimum alginate concentration is 2% and chitosan is 0.5% [9].

**Figure 2:**
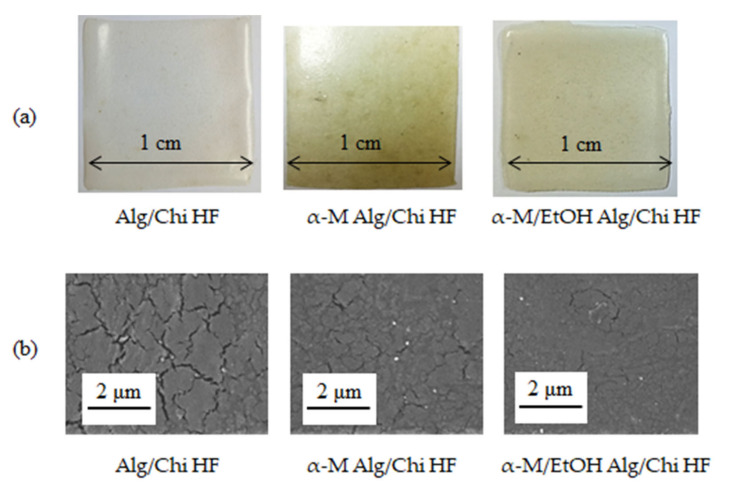
Physical appearances of Alg/Chi HF, α-M Alg/Chi HF, and α-M/EtOH Alg/Chi HF. Macroscopic (**a**) and microscopic (**b**) appearances.

Alg/Chi HF film seems transparent and homogenous, while α-M Alg/Chi HF films are yellow and inhomogenous, while α-M/EtOH Alg/Chi HF film appears to be yellow in color and transparent (Figure 2a).

Based on the results obtained from the SEM instrumentation analysis, there are differences between the three formulas where in formula 1, the Alg/Chi HF has a slightly hollow surface. The same thing is also seen in formula 2, where the α-M Alg/Chi HF still shows α-M granules on the surface of the film, which indicates that the α-M is not completely dissolved in the carrier, whereas in formula 3, the α-M/EtOH Alg/Chi HF is the best cross-section of the three because it has a smooth surface. In α-M/EtOH Alg/Chi HF, the α-M was completely dissolved in ethanol and dispersed in Alg/Chi HF homogenous (Figure 2b). The results of SEM analysis are correlated with the macroscopic appearance of the hydrogel films, where in the α-M Alg/Chi HF, there were still insoluble granules (Figure 2a).

### 3.2. Physicochemical Properties of Alg/Chi HF, α-M Alg/Chi HF, and α-M/EtOH Alg/Chi HF

Before characterization using SEM and XRD, it is necessary to determine the physical properties of the hydrogel film, such as thickness, weight, and pH of the produced hydrogel.

**Figure 3:**
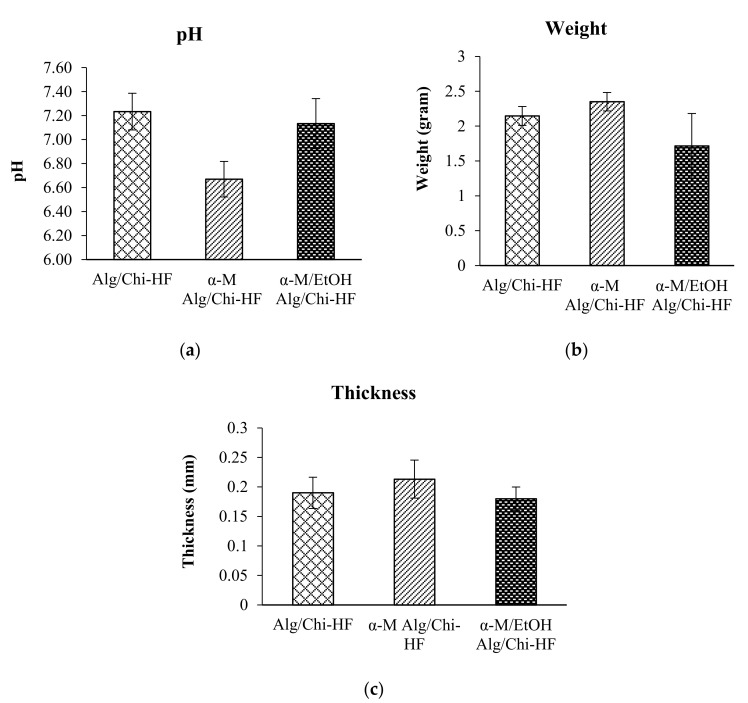
The thickness (**a**), weight (**b**), and pH (**c**) of Alg/Chi HF, α-M Alg/Chi HF, and α-M/EtOH Alg/Chi HF. The results are expressed as the mean ± SD (*n* = 3).

Based on the results of thickness measurements, we found that the average thickness of Alg/Chi HF, α-M Alg/Chi HF, and α-M/EtOH Alg/Chi HF was 0.19 ± 0.03 mm, 0.21 ± 0.03 mm, and 0.18 ± 0.02 mm, respectively (Figure 3a), while the average weight of Alg/Chi HF is 2.15 ± 0.14 g, α-M Alg/Chi HF is 2.35 ± 0.13 g, and α-M/EtOH Alg/Chi HF is 1.72 ± 0.46 g (Figure 3b). Based on the statistical results, there is no significant difference between Alg/Chi HF, α-M Alg/Chi HF, and α-M/EtOH Alg/Chi HF (α = 0.05). The statistical analysis was performed using the Scheffe test to compare each of the formulations.

The pH of Alg/Chi HF, α-M Alg/Chi HF, and α-M/EtOH Alg/Chi HF were 7.2 ± 0.15, 6.6 ± 0.15, and 7.3 ± 0.21, respectively (Figure 3c). Based on the statistical test results, there is no significant difference between Alg/Chi HF, α-M Alg/Chi HF, and α-M/EtOH Alg/Chi HF (α = 0.05).

### 3.3. Swelling Ratio and Mucoadhesive Time

**Figure 4:**
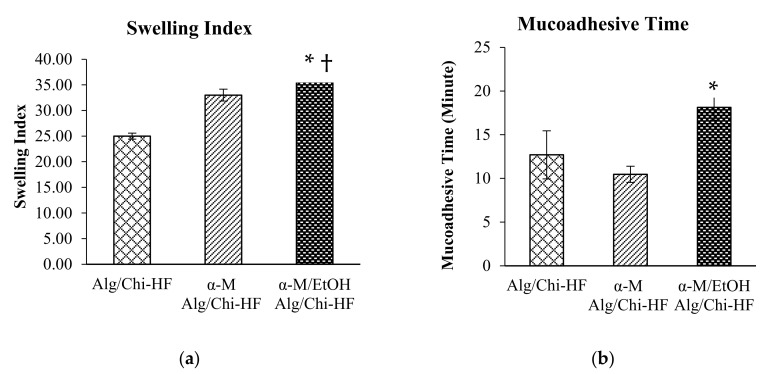
Swelling index (**a**) and mucoadhesive time (**b**) of Alg/Chi HF, α-M Alg/Chi HF, and α-M/EtOH Alg/Chi HF. The results are expressed as the mean ± SD (*n* = 3). † *p* < 0.0001 and * *p* < 0.05 compared to Alg/Chi-HF.

The first stage of the swelling ratio test before calculating the weight was soaking the hydrogel film in PBS. The swelling ratio of Alg/Chi HF is 25 ± 0.58%, α-M Alg/Chi HF is 33 ± 1.15%, and α-M/EtOH Alg/Chi HF is 37 ± 0.29% (Figure 4a). Based on the statistical results, there are significant differences between the α-M/EtOH Alg/Chi HF against Alg/Chi HF(^†^ *p* < 0.0001) and α-M Alg/Chi HF (* *p* < 0.05). The statistical analysis was done by using the Scheffe test to compare each of the formulations (Figure 4a). Alg/Chi HF, α-M Alg/Chi HF, and α-M/EtOH Alg/Chi HF were able to swell more than 20%; therefore, they can be used as dosage forms for therapy.

Evaluation of mucoadhesive time aimed to determine the time required by α-mangostin hydrogel film to adhere to the mucosal layer. The statistical analysis revealed that there is no significant difference between the Alg/Chi HF against α-M Alg/Chi HF, but there is a significant difference between α-M/EtOH Alg/Chi HF compared to Alg/Chi HF and α-M Alg/Chi HF (* *p* < 0.05).

The average results of the mucoadhesive time of Alg/Chi HF, α-M Alg/Chi HF, and α-M/EtOH Alg/Chi HF are 12.7 ± 2.75 min, 10.2 ± 0.93 min, and 19.3 ± 1.46 min, respectively (Figure 4b). It is known that the best mucoadhesive percent is in the α-M/EtOH Alg/Chi HF for 19.3 ± 1.46 min. α-M/EtOH Alg/Chi HF adhere in a longer time compared to α-M Alg/Chi HF and Alg/Chi HF. Therefore, there is a significant difference between α-M/EtOH Alg/Chi HF against Alg/Chi HF with * *p* < 0.05 (*n* = 3).

### 3.4. Characterization of Alg/Chi HF, α-M Alg/Chi HF, and α-M/EtOH Alg/Chi HF

**Figure 5:**
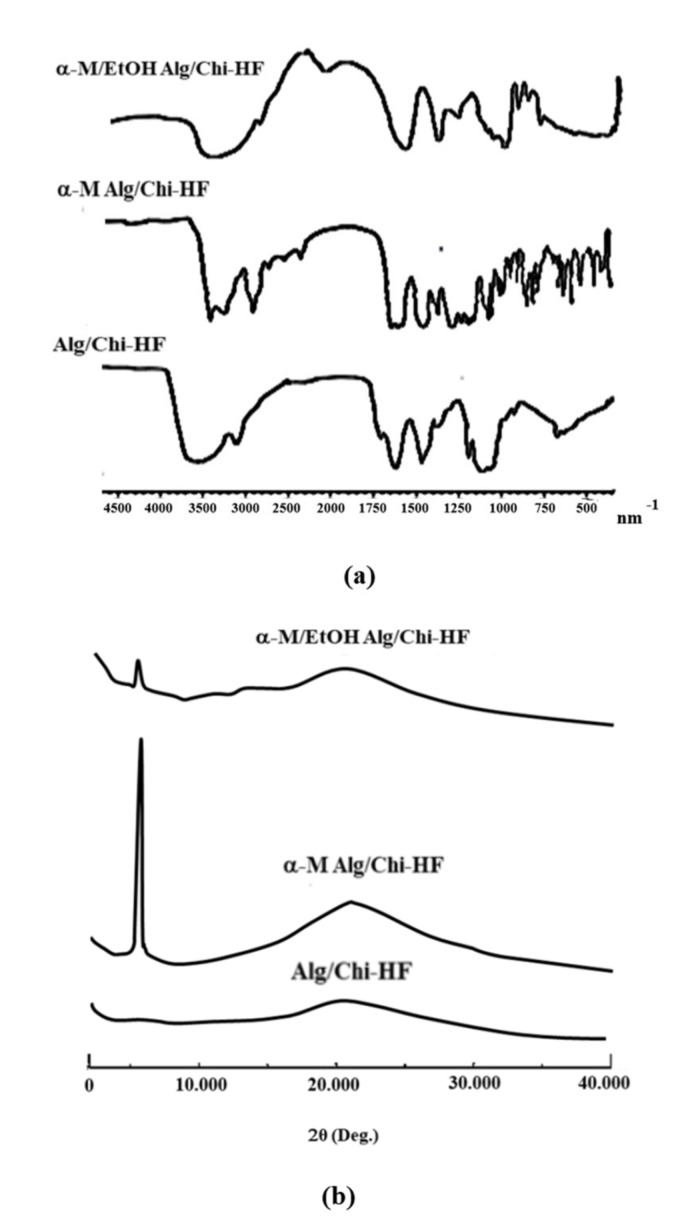
(**a**) FTIR spectra; and (**b**) X-ray patterns of Alg/Chi HF, α-M Alg/Chi HF, and α-M/EtOH Alg/Chi HF.

The FTIR results from the three formulas show that there are strong and wide peaks at wave numbers of 3000–3500 nm^−1^, where the strong peaks are shown at wave numbers of 3300 nm, indicating the presence of OH group. In the wave number of 2500–2700 nm^−1^, there is a specific peak and has a strong C-H and C=H corresponding to aromatic ring peak at a wave number of 690–900 nm^−1^ but varies in the 1500–1600 nm^−1^ (Figure 5a).

XRD analysis was conducted to determine the shape characteristics of the α-mangostin hydrogel film with an alginate–chitosan combination polymer. The crystallinity of Alg/Chi HF, α-M Alg/Chi HF, and α-M/EtOH Alg/Chi HF was investigated using XRD (Figure 5b). XRD results on the analysis of the hydrogel films of Alg/Chi HF, α-M Alg/Chi HF, and α-M/EtOH Alg/Chi HF reveal the diffraction patterns of the crystalline and amorphous systems which characterize these materials. The standard diffraction pattern of α-M shows a high-intensity peak indicating that α-M is a crystalline form [23]. The alginate and chitosan polymers have a standard amorphous pattern (no sharp peaks) [9].

### 3.5. In Vitro Drug Release

**Figure 6:**
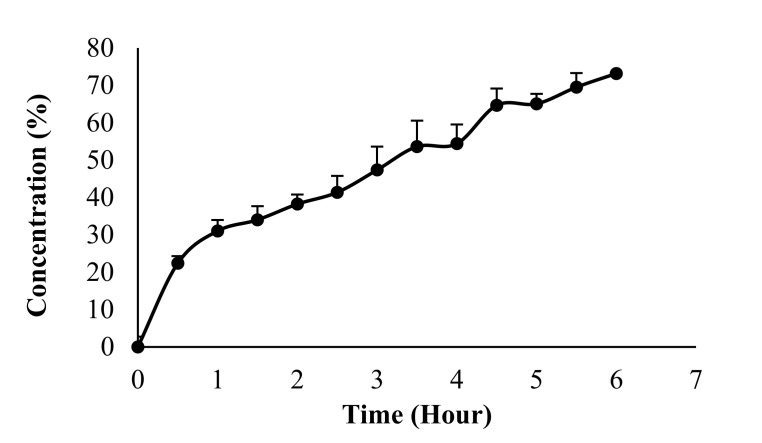
In vitro drug release of a-M/EtOH Alg/Chi-HF. Each value represents the mean ± SD of three experiments.

Based on the performed experiments, 22.40 ± 2.75% of α-M was released after 30 min. The cumulative drug release reached over 50% after 3.5 h (53.59 ± 6.24%). After 6 h, the cumulative drug release was 73.16 ± 3.77% (Figure 6). This can be explained as the hydrogel film consisting of polymers as matrix of the active substance, which in turn extends the release time [19].

### 3.6. In Vitro Viability Study

**Figure 7:**
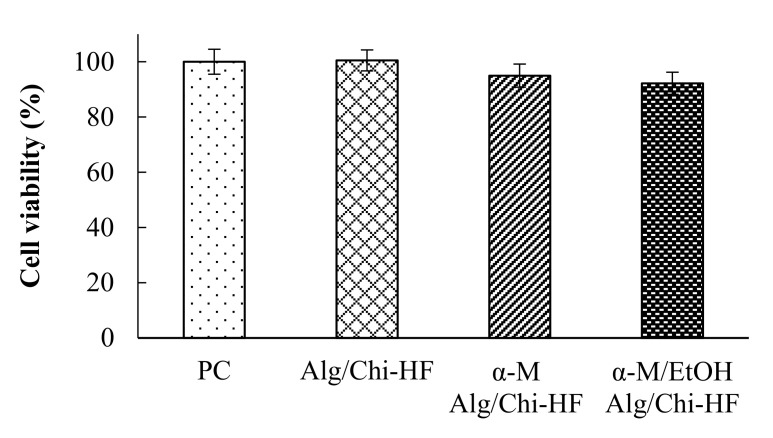
In vitro cell viability of α-M/EtOH Alg/Chi-HF. In vitro proliferation was carried out using NIH/3T3 cells. Each value represents the mean ± SD of three experiments.

After 24 h incubation, the hydrogel films of Alg/Chi HF, α-M Alg/Chi HF, and α-M/EtOH Alg/Chi HF showed a cell viability of 100.50 ± 3.79%, 94.94 ± 4.21%, and 92.19 ± 4.02%, respectively. The results can be seen in Figure 7. Therefore, all of the preparation had good tolerability. Consequently, hydrogel films are considered to have a good level of safety [1].

### 3.7. In Vivo Wound Healing Study

**Figure 8:**
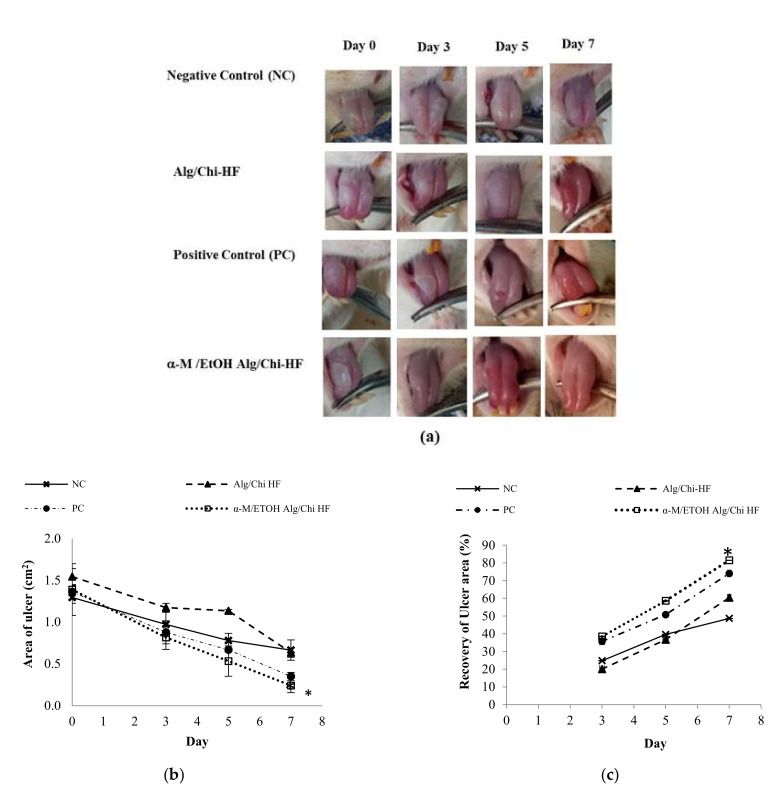
In vivo wound-healing study of a-M/EtOH Alg/Chi-HF (**a**). The decrease in ulcer area (**b**). The percentage recovery of ulcer area (**c**). Each value represents the mean ± SD of three experiments. On the 7th day, * *p* < 0.05, compared to the NC and Alg/Chi HF.

Based on the tests that have been carried out, the results are in accordance with what is depicted in Figure 8. Compared to the positive (PC) and negative control (NC) groups, the percentage of healing in the α-M/EtOH Alg/Chi HF group was higher. The recovery of ulcer area in the NC group on the 3rd day was 24.82 ± 0.11%, on the 5th day was 39.62 ± 0.09% and on the 7th day was 48.69 ± 0.08%. While the recovery of ulcer area in PC on the 3rd day was 35.71 ± 0.07%, on the 5th day, it was 50.81 ± 0.03%, and on the 7th day, it reached 74.05 ± 0.05%. The recovery of ulcer area in the Alg/Chi HF group on the 3rd day was 20.08 ± 0.07%, on the 5th day was 36.68 ± 0.08%, and on the 7th day was 60.40 ± 0.05%. The recovery of ulcer area in the α-M/EtOH Alg/Chi HF group on the 3rd day was 38.51 ± 0.20%, on the 5th day was 58.62 ± 0.20%, and on the 7th day was 81.47 ± 0.09%. Based on the description of the existing results, it is known that the group that has the highest recovery of ulcer area is the α-M/EtOH Alg-/Chi HF group. On the 7th day, the percentage of healing reached more than 80%. The results obtained are shown in Figure 8b,c.

### 3.8. Histopathological Analysis

**Figure 9:**
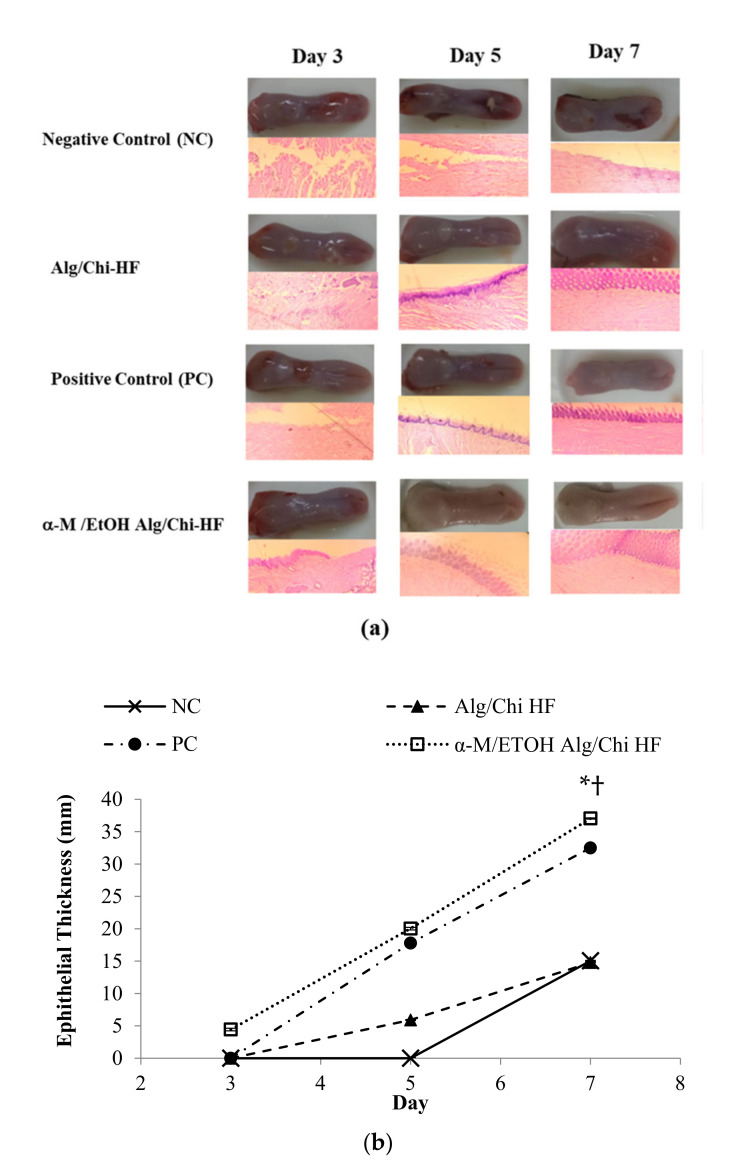
Histopathology study of tongue from treatment group of in vivo study (**a**). The increase in epithelial layer thickness in 7 days (**b**). Each value represents the mean ± SD of three experiments., * *p* < 0.05, compared to the PC, † *p* < 0.0001, compared to Alg/Chi HF and NC.

Data from observations of histopathological studies in four groups of experimental animals showed good results in healing (Figure 9a). The parameter used in analyzing the results of histopathological studies is the thickness of the epithelial layer formed. The repair of the connective tissue layer started after the 5th day in the negative control. On the 7th day, the repair of connective tissue in the mucosal layer rather than the epithelial thickening is seen in Figure 9a.

In the current study, the epithelial thickness was measured using ImageJ software and plotted in a linear graph (Figure 9b). Furthermore, the slope was calculated to analyze the faster healing group. α-M/EtOH Alg/Chi HF showed the fastest healing process based on the clope calculation.

Re-epithelization is one of the ulcer healing indicators. The epithelial thickness increased in each group, but in the α-M/EtOH Alg/Chi HF group, the epithelia started to form on the 3rd day, but in Alg/Chi HF and PC, the healing started from the 5th day. In the NC, the epithelia were formed on the 7th day, correlated with the normal healing oral ulcer, which was reported to spontaneously heal in 5–7 days [24,25].

The repairing of the oral epithelia was promoted by tissue formation, resulting in an increase in epithelial thickness. Chi is one of the mucoadhesive polymers that has an ability for tissue regeneration, which eventually resulted in the increase in Alg/Chi HF-treated group healing process [26].

## 4. Discussion

α-M has poor solubility in water; therefore, the α-M Alg/Chi HF revealed an inhomogenous distribution of α-M in the hydrogel films. To obtain homogenous concentration α-M in the hydrogel film, we tried to dissolve α-M in ethanol as an appropriate solvent before mixing with HF base [17,27], resulting in homogenous film formation (Figure 2a).

The prepared hydrogel films are composed of combinations of two polymers, namely alginate and chitosan. Alginate and chitosan are a combination of polymers that form cross bonds through cross-linking. Alginate was successfully used as a polymer in hydrogel film formulations and in nanoparticle and gel polymers. Besides alginate, chitosan is also used as a polymer in this study [20]. Chitosan has been widely used as a biomaterial or polymer for hydrogel systems [21]. Based on the in silico studies, alginate and chitosan combinations show an interaction with each other through cross-linking (alginate–chitosan) and with α-M, in which α-M acts as a receptor and alginate as a donor [9]. Other additional ingredients are plasticizers, such as propylene glycol and glycerin. Plasticizers are used to increase the flexibility of the hydrogel film and enhance the absorption of mucous membranes, and as a preservative. Hydrogel films tend to be oily when the propylene-glycol and glycerin concentrations are more than 5% and 2%, respectively, and will produce a rigid film when below these concentrations. Thus, it can be concluded that the optimum concentration of propylene-glycol is 5% and glycerin is 2%. The hydrogel film preparation scheme can be seen in Figure 2.

Measurement of pH aims to determine whether the preparation produced is in accordance with the tolerance of mucosal acceptance, which is directly related to the comfort while using the preparation. Based on a literature review, the pH of mucosa ranged from 6.5 to 7.5, which corresponds to the salivary pH [28]. To avoid any possible mucosal irritation, the pH of the formulation must be in accordance with the pH of the mucosa. The pH of the three formulas is still within the mucosal pH range; thereby, it can be said that it is safe and non-irritating when used. The critical pH of saliva is 5.5 because if the preparation is below this pH, it may cause the devastation of the calcium ion, which leads dental caries [29].

The evaluation of thickness and weight showed no significant difference between the thickness of the three hydrogel film formulas. Therefore, these results demonstrate that all preparation met the requirements for the thickness of the hydrogel, 0.1–1 mm [16].

A swelling ratio was performed to find out the swelling characteristics of α-mangostin hydrogel film with alginate–chitosan polymers. The three formulas showed that the swelling ratio is more than 20% and considered that these formulas have the good swelling ability and can be used in several pharmaceutical dosage forms. The swelling ratio is the ability of the polymer to expand and open the polymer net (pores) so that the solvent can penetrate into the polymer matrix and maintain a diffusion system [24]. The swelling ratio of the polymer is directly proportional to the ability of polymers to allow the diffusion of the actives. A polymer is said to have a good system when the swelling index is more than 20% [23].

Hydrogel films are composed of three-dimensional structured polymers that can absorb water molecules. The unique feature of this dosage form is to absorb water and swell accordingly [30]. The Swelling Index of the preparations correlated with the ability of polymers to release the active ingredients [30].

The combination of Alg and Chi improved the mucoadhesiveness of the film through the formation of a mechanical bond of the HF with the mucosa. The presence of Chi increases the mucoadhesive time because of the interaction between the positive charges of Chi with sialic acid on the surface of the mucosa [26,31].

Mucoadhesive preparations can adhere to the mucus membranes for a longer period of time so as to increase contact time and increase the bioavailability of the active substance in the mucous membrane. Alginate is a polymer that has good mucoadhesive properties with a swelling ratio of more than 20%. The increase in the concentration of sodium alginate is directly proportional to the stickiness [26]. Besides alginate, chitosan is used as a co-polymer that can increase the adhesion to the mucosal membrane. Chitosan is a polymer originating from shrimp shells or other animals containing kitin. The combination of both polymers can enhance the mucoadhesive power to the mucous membranes, thus, reducing the frequency of administration.

As shown in Figure 3a, FTIR results of Alg/Chi HF, α-M Alg/Chi HF, and α-M/EtOH Alg/Chi HF showed a strong absorption around 3500 nm^−1^, signifying the N-H or amine bonds which are characteristic to chitosan, while a broad band at 3300 nm^−1^ indicates the presence of hydrogen bond at hydroxyl groups. The weak absorption around 2900 nm^−1^ is given by C-H with sp^3^ hybridization. Thus, it concluded that these three formulas have both functional groups in alginate and chitosan. The stretching vibration at 1500–1600 nm^−1^ is shown to be unconjugated C=C of α-M in α-M Alg/Chi HF and α-M/EtOH Alg/Chi HF, while aromatic ring shown to be strong absorption at 690–900 nm^−1^, indicates the presence of α-M in the films [23,32].

The XRD pattern of Alg/Chi HF showed an amorphous diffractogram pattern (the absence of high-intensity peaks indicates that alginate and chitosan had good solubility). At 5.89°, α-M Alg/Chi HF displayed a high-intensity peak [23], indicating that α-mangostin is in a crystalline form [17,33,34,35]. In α-M/EtOH Alg/Chi HF, the peak still appears at the same 2θ (Figure 3b); the peak has a lower intensity [7], which indicates that α-M was coated in Alg/Chi HF.

It has been reported that α-M/EtOH Alg/Chi HF a was easily synthesized into the alginate-chitosan hydrogel film after α-mangostin was dissolved mechanistically inside the polymers with the usage of ethanol [7].

In vitro drug release was performed to evaluate the percentage of active substances that come out of the polymer based on units of time. Q value determines the drug release mechanism. When Q approaches 0.5, then the release mechanism follows Fick’s First Law of Diffusion, of which the diffusion value is 0.5 < Q < 1. Based on the testing carried out, the results are in accordance with Figure 6.

The presence of Alg and Ch increases the release of α-M. The presence of Alg in Chi films leads to the relaxation of electrostatic interaction of amino group in Chi and carbonyl group in Alg [36,37]. The stronger interaction increased the attachment time of α-M/EtOH Alg/Chi HF at the mucous layer, resulting in the extended drug time release [7]. This is also because the hydrogel film contains polymers that can absorb the active substance and release it slowly.

In the fabrication of hydrogel films as local site delivery, it is necessary that these hydrogels don’t cause systemic toxicity.

Based on the tests that have been carried out, the results are in accordance with what is depicted in Figure 8. With reference as illustrated in Figure 8, the greatest recovery of ulcer area was associated with decreases in ulceration area until the end of this experiment. The highest recovery of ulcer area was that of the α-M/EtOH Alg/Chi HF-treated group correlated with the faster healing activity [38].

Alg has mucoadhesive properties on the mucous layer but dissolves rapidly [7]. The addition of Chi increases the mucoadhesiveness of α-M/EtOH Alg/Chi HF at the mucosal surface, which forms a protective layer covering the oral ulcer, avoids the effect of food and drinks, etc. Thus, the combination of these two polymers is able to provide coverage to the ulcer for a longer time [26]. The swelled Alg/Ch HF covered the ulcer at the mucous layer for an extended time, thus giving enough time for α-M to exert its anti-inflammatory effect. The anti-inflammation effect of α-M occurs by the inhibition of inflammation mediator release such as COX-2, IL-1, IL-6, TNF-α dan NO or blocking of the translocation of NFkB from the nucleus to the cytoplasm [39,40].

In addition, the increasing epithelial thickness and improved ulcer healing can be attributed to the anti-inflammatory effect of α-M, which suppresses the production of TNF-α and inhibits cytokine synthesis. This activity induces re-epithelization and play a role in the restoration of the mucous layer [41].

## 5. Conclusions

α-Mangostin hydrogel film has the potential to be used in the treatment of recurrent aphthous stomatitis (RAS). The physicochemical characteristics of α-M/EtOH Alg/Chi HF film, including pH, thickness, weight, and swelling ratio, met the requirements for oral preparation. Furthermore, the mucoadhesive study demonstrates that α-M/EtOH Alg/Chi HF has the highest mucoadhesive time when compared to other preparations. The α-M/EtOH Alg/Chi HF formula revealed a dispersed α-mangostin across the hydrogel film, which demonstrated a favorable healing response on the seventh day. This is strongly related to the histology investigation, which shows significant epithelial growth. Based on these findings, α-M/EtOH Alg/Chi HF will be the new RAS treatment preparation type.

## Figures and Tables

**Table 1:** Preparation of α-M/EtOH Alg/Chi HF.

Alg/Chi HF	Alginate Sodium (g)	Chitosan (g)	Propylene Glycol (%*w*/*v*)	Glycerin (%*w*/*v*)	α-M (μg)	Ethanol (μL)	Acetic Acid (mL)	Distilled Water (mL)
Alg	0.5	-	-	-	-	-	-	25
Chi	-	0.1	-	-	-	-	0.5	24.5
Alg/Chi	0.5	0.1	5	2	-	-	0.5	49.5
α-M	0.5	0.1	5	2	250	-	0.5	49.5
α-M/EtOH	0.5	0.1	5	2	250	10	0.5	49.5

## Abbreviations

Alg	Alginate
α-M	Alpha mangostin
Chi	Chitosan
COX	Cyclooxigenase
EtOH	Ethanol
FTIR	Fourier transform infrared spectroscopy
HF	Hydrogel film
IL	Interleukin
PBS	Phosphate-buffered saline
PC	Positive control
NC	Negative control
NFkB	Nuclear factor kappa Beta
NO	Nitric oxide
RAS	Recurrent aphthous stomatitis
SD	Standard deviation
SEM	Scanning electron microscopy
TNF-α	Tumor mecrosis factor-alpha
XRD	X-ray diffraction
